# Comparing in Cylinder Pressure Modelling of a DI Diesel Engine Fuelled on Alternative Fuel Using Two Tabulated Chemistry Approaches

**DOI:** 10.1155/2014/534953

**Published:** 2014-10-28

**Authors:** Claude Valery Ngayihi Abbe, Robert Nzengwa, Raidandi Danwe

**Affiliations:** ^1^Faculty of Industrial Engineering, P.O. Box 2701, University of Douala, Douala, Cameroon; ^2^National Advanced School of Engineering, P.O. Box 337, University of Yaounde, Yaounde, Cameroon; ^3^Higher Institute of the Sahel, P.O. Box 46, University of Maroua, Maroua, Cameroon

## Abstract

The present work presents the comparative simulation of a diesel engine fuelled on diesel fuel and biodiesel fuel. Two models, based on tabulated chemistry, were implemented for the simulation purpose and results were compared with experimental data obtained from a single cylinder diesel engine. The first model is a single zone model based on the Krieger and Bormann combustion model while the second model is a two-zone model based on Olikara and Bormann combustion model. It was shown that both models can predict well the engine's in-cylinder pressure as well as its overall performances. The second model showed a better accuracy than the first, while the first model was easier to implement and faster to compute. It was found that the first method was better suited for real time engine control and monitoring while the second one was better suited for engine design and emission prediction.

## 1. Introduction

The modeling of internal combustion engines has been largely developed during past years. For this purpose, a multitude of industrial codes dedicated to the simulation of the engines is available on market (GT-Power, Diesel-RK, Ricardo-Wave, Fluent, etc.). These codes, despite the fact that they can be useful for predicting engine performances, are expensive for third world's university laboratory and their source codes are practically impossible to modify to implement new model or functions.

When modeling compression engine different approaches can be used with different level of complexity, such as thermodynamic 0D models, quasi-dimensional multizone models, and computational fluid dynamics (CFD) models [1, 2].

For this study a quasi-dimensional approach was selected; these models allows to compute efficient, economic, and fast calculations of engine performances as a function of different engine parameters. Using these types of models we can compute the different stages of a diesel engine cycle as in compression, injection, ignition delay, and combustion and exhaust stage.

The evaluated models in this study will be evaluated in terms of accuracy and speed of calculation.

## 2. Governing Equations

### 2.1. Fuel Injection and Vaporization Model

For both models the fuel spray characteristic is modeled using the phenomenological model of Razleytsev [3] and Lyshevsky [4]. That model has been used and implemented in the so called RK-model [5] and its results. The main aim of this part of the model is to determine the finesse of the atomized fuel from the injector nozzle. The finesse of atomization is characterized by the calculated Sauter mean diameter of fuel droplet. The model is a simple sequence of calculated parameters and is structured as follows.

First the average outflow velocity of fuel from the injector is calculated in m/s by

(1) $$\begin{array}{c} U_{0}=\frac{24q_{c}\text{RPM}}{0.75\rho_{f}\pi d_{c}^{2}i_{c}\varphi_{\text{inj}}}, \end{array}$$

where *q*_*c*_ is the cyclic fuel supply in Kg/cycle; RPM is the crankshaft rotational speed in rpm; *ρ*_*f*_ is the density of the fuel in kg/m^3^; *d*_*c*_ is the diameter of the injector hole in mm; *φ*_inj_ is the duration of injection in degree of rotation of the crankshaft.

The criterion *M* characterizing the relationship between the surface tension force, inertia, and viscosity is calculated as follows:

(2) $$\begin{array}{c} M=\frac{\mu_{f}^{2}}{\left(d_{c}\rho_{f}\sigma_{f}\right)}, \end{array}$$

where *μ*_*f*_ is the coefficient of dynamic viscosity of the fuel at a temperature of 323 K in Pa·s; *σ*_*f*_ is the coefficient of surface tension of the fuel at a temperature of 323 K in N/m.

Weber number characterizing the relationship between surface tension force and inertia is determined as

(3) $$\begin{array}{c} W_{e}=\frac{U_{0}^{2}\rho_{f}d_{c}}{\sigma_{f}}. \end{array}$$

The density of the charge at the end of the compression before the TDC is calculated as follows:

(4) $$\begin{array}{c} \rho_{\text{air}}=\frac{\mu_{\text{air}}M_{\nu }}{V_{c}}, \end{array}$$

where *μ*_air_ = 28.9 Kg/Kmole is the molecular mass of air; *ρ*_air_ is the density of air.

We then determine the fuel/air density ratio by

(5) $$\begin{array}{c} \rho =\frac{\rho_{\text{air}}}{\rho_{f}}. \end{array}$$

Finally the Sauter mean diameter of the atomized fuel is calculated in micron by

(6) $$\begin{array}{c} d_{32}=\frac{10^{6}E_{32}d_{c}M^{0,0733}}{{\left(\rho W_{e}\right)}^{0,266}}, \end{array}$$

where *E*_32_ is an empirical factor depending on designs of the injector, whose recommended value is 1.7.

At this point the finesse of the pulverized fuel is determined and we can see it mainly depends on physical properties of the fuel such as viscosity and density. The next step of the model is to determine the kinetic of combustion of the atomized fuel, whose kinetic will be dependent of the earlier found Sauter mean diameter.

### 2.2. Model of the Kinetic of Combustion of the Atomized Fuel

This part of the elaborated model will permit us to determine parameters of the process such as the evaporation rate of the atomized fuel, its ignition delay, and the duration of combustion.

Pressure in the cylinder at the end of the compression before the TDC is

(7) $$\begin{array}{c} P_{c}=P_{0}\varepsilon^{1,37}, \end{array}$$

where *P*_0_ is the reference pressure which in our case corresponds to the inlet manifold pressure.

We then determine the theoretical constant of evaporation of the fuel by

(8) $$\begin{array}{c} b_{\text{it}}=\frac{K}{d_{32}^{2}}, \end{array}$$

where *K* = 1/*P*_*c*_ · 10^6^ is the evaporation constant related to cylinder pressure in m^2^/s

(9) $$\begin{array}{c} \tau_{ev}=\frac{A_{z}}{\left(b_{\text{it}}\lambda^{0,6}\right)}, \end{array}$$

where *A*_*z*_ is a constant characterizing the duration of evaporation of large drops in diesel engine in 1/s and its value is given as  2.4 in [3] but Kuleshov [5] used it as weight coefficient that can be varied in order to match experimental data; *λ* is the coefficient of excess air.

The full duration of combustion is then calculated as

(10) $$\begin{array}{c} \phi_{z}=\phi_{\text{inj}}-\text{ID}+6\text{RPM}\tau_{ev}, \end{array}$$

with ID being the ignition delay computed using the formula of Hardenberg and Hase [6] in both models

(11) ID=[0.36+0.22U−p]  ×exp⁡[Ea·(1RTimεnc−1−117190)+(21.2Pimεnc−12.4)0.63].

The value of the apparent activation energy *E*_*a*_ in this correlation is given by *E*_*a*_ = 618840/(*CN* + 25); however, this value was computed for the particular experiment reported in [6] and needs to be corrected in order to be used for different experimental conditions. In order to match experimental ignition delay values, especially for biodiesel fuel, the method proposed by Aghav et al. [7], where the value 618840 can be varied from the initial 618840 to higher values, was used.

### 2.3. Heat Release and Heat Transfer Laws

For the two models, we used a double Wiebe function [8] to model the heat release rate and the Woschni [9] law for heat transfer simulation.

## 3. Combustion Models

### 3.1. Method 1: Combustion Model Based on Borman and Krieger [10]

This method is a single zone model based on the first law of thermodynamics, mass conservation, and ideal gas laws. Following that we can write the governing equation for calculating variation of in-cylinder pressure with respect to crank angle as [11]

(12) $$\begin{array}{c} \frac{dQ_{\text{inj}}-dQ_{\text{loss}}}{d\theta }=\frac{\gamma }{\gamma -1}P\frac{dV}{d\theta }+\frac{1}{\gamma -1}V\frac{dP}{d\theta }. \end{array}$$

The Krieger and Bormann algorithm is based on polynomial fitting constant to compute the adiabatic index (ratio of specific heat) as function of crank angle. The fitting coefficients can be found in the Appendix.

The internal energy of combustion products at equilibrium for a reaction between air and a hydrocarbon CnH2n is given as

(13) $$\begin{array}{c} u=\frac{A-B}{\lambda +u_{\text{corr}}}, \end{array}$$

where

(14) $$\begin{array}{c} A=a_{1}T+a_{2}T^{2}+a_{3}T^{3}+a_{4}T^{4}+a_{5}T^{5};\\ B=b_{0}+b_{1}T+b_{2}T^{2}+b_{3}T^{3}+b_{4}T^{4}; \end{array}$$

*u*_corr_ = *c*_*u*_exp⁡(*D*_*λ*_ + *E*_*λ*_ + *F*_*PTλ*_) is the correction factor for the internal energy accounting for dissociation

(15) $$\begin{array}{c} D_{\lambda }=d_{0}+d_{1}\cdot \lambda^{-1}+d_{3}\lambda^{-3};\\ E_{\lambda }=\frac{\left(e_{0}+e_{1}\lambda^{-1}+e_{3}\lambda^{-3}\right)}{T};\\ F_{PT\lambda }=\left[f_{0}+f_{1}\lambda^{-1}+f_{3}\lambda^{-3}+\frac{\left(f_{4}+f_{5}\lambda^{-1}\right)}{T}\right]\log\left(f_{6}\cdot P\right). \end{array}$$

The gas constant is computed as

(16) $$\begin{array}{c} R=0.287+\frac{0.020}{\lambda }+R_{\text{corr}}, \end{array}$$

with *R*_corr_ = *c*_*r*_exp⁡(*r*_0_log⁡*λ* + ((*r*_1_ + *r*_2_/*T*) + *r*_3_log⁡(*f*_6_ *P*))/*λ*).

The adiabatic index (ratio of specific heat) is computed as

(17) $$\begin{array}{c} \gamma =1+\frac{R}{c_{v}}, \end{array}$$

with *c*_*v*_ = ∂*u*/∂*T*.

### 3.2. Method 2: Combustion Model Based on Olikara and Bormann Method [12, 13]

This model is a two-zone thermodynamic model where the thermodynamics properties of combustion products and fuel are determined using polynomial curve fitted data from the Chemkin [14] or JANAF [15] tables and are given by

(18) $$\begin{array}{c} \frac{c_{p}}{R}=a_{1}+a_{2}T+a_{3}T^{2}+a_{4}T^{3}+a_{5}T^{4};\\ \frac{h}{RT}=a_{1}+\frac{a_{2}}{2}T+\frac{a_{3}}{4}T^{2}+\frac{a_{4}}{4}T^{3}+\frac{a_{5}}{5}T^{4}+a_{6}\frac{1}{T};\\ \frac{s}{R}=a_{1}\ln T+a_{2}T+\frac{a_{3}}{2}T^{2}+\frac{a_{4}}{3}T^{3}+\frac{a_{5}}{4}T^{4}+a_{7}. \end{array}$$

The in-cylinder pressure and temperature of burned and unburned gases derivative can be calculated following Ferguson's algorithm [13, 16] by

(19) dPdθ=A+B+CD+E;dTbdθ=−h(πb2/2+4V/b)x1/2Tb−TwvmcPbx +vbcPb(∂ln⁡vb∂ln⁡Tb)(dPdθ) +hu−hbxcPb[dxdθ−x−x2Cω];dTudθ=−h(πb2/2+4V/b)x1/2Tu−TwvmcPux +vucPu(∂ln⁡vu∂ln⁡Tu)(dPdθ) +hu−hbxcu[dxdθ−x−x2Cω],

where

(20) A=1m(dVdθ+VCω);B=h(dV/dθ+VC/ω)ωm ×[vbcPb∂ln⁡vb∂ln⁡Tbx1/2Tb−TwTb   +vucPu∂ln⁡vu∂ln⁡Tu(1−x1/2)Tu−TwTu];C=−vb−vudxdθ−vb∂ln⁡vb∂ln⁡Tbhu−hbcPbTb[dxdθ−(x−x2)Cω];D=x[vb2cPbTb(∂ln⁡vu∂ln⁡Tu)2+vbP∂ln⁡vb∂ln⁡P];E=1−x[vu2cPuTu(∂ln⁡vu∂ln⁡Tu)2+vuP∂ln⁡vu∂ln⁡P].

The equations of state of the mixture are given by

(21) $$\begin{array}{c} \frac{du}{d\theta }=\left(c_{P}-\frac{Pv}{T}\frac{\partial \ln v}{\partial \ln T}\right)\frac{dT}{d\theta }-\left(v\left(\frac{\partial \ln v}{\partial \ln T}+\frac{\partial \ln v}{\partial \ln P}\right)\right)\frac{dP}{d\theta };\\ \frac{dv}{d\theta }=\frac{v}{T}\frac{\partial \ln v}{\partial \ln T}\frac{dT}{d\theta }-\frac{v}{P}\frac{\partial \ln v}{\partial \ln P}\frac{dP}{d\theta };\\ \frac{ds}{d\theta }=\left(\frac{c_{P}}{T}\right)\frac{dT}{d\theta }-\frac{v}{T}\frac{\partial \ln v}{\partial \ln T}\frac{dP}{d\theta }, \end{array}$$

with (∂*h*/∂*T*)_*P*_ = *c*_*P*_.

The combustion products are evaluated assuming that they are in equilibrium at a given temperature and pressure. Using Olikara and Borman [12] method we find combustion products mole fractions and we can then find the thermodynamic property of the mixture as in entropy, specific volume, enthalpy, and internal energy.

Considering the combustion reaction of a hydrocarbon

(22) CαHβOγNδ+asϕ(O2+3.76N2) ⟶n1CO2+n2H2O+n3N2+n4O2+n5CO   +n6H2+n7H+n8O+n9OH+n10NO

we then have

(23) $$\begin{array}{c} \text{C}:\alpha =\left(y_{1}+y_{5}\right)T_{m};\\ \text{H}:\beta =\left(2y_{2}+2y_{6}+y_{7}+y_{9}\right)T_{m};\\ \text{O}:\gamma +\frac{2a_{s}}{\phi }=\left(2y_{1}+y_{2}+2y_{4}+y_{5}+y_{8}+y_{9}+y_{10}\right)T_{m};\\ \text{N}:\delta +\frac{2\cdot 3.76a_{s}}{\phi }=\left(2y_{3}+y_{10}\right)T_{m}; \end{array}$$

*y*_*i*_ is the mole fraction of the *i*th combustion specie at equilibrium and *T*_*m*_ the total number of mole. Following that we can write ∑_*i*=1_^10^ *y*_*i*_ − 1 = 0.

The system of equation comprises 10 unknowns and 4 equations; we then need 6 complementary equations in order to be able to solve the system. For that we introduce six gas phase equilibrium reactions constant

(24) 12H2⟺HKp,1=y7p1/2y61/212O2⟺OKp,2=y8p1/2y41/212H2+12O2⟺OHKp,3=y9y41/2y61/212O2+12N2⟺NOKp,4=y10y41/2y31/4H2+12O2⟺H2OKp,5=y2y41/2y6p1/2CO+12O2⟺CO2Kp,6=y1y41/2y5p1/2

with *p* representing the pressure at which the reaction occurs in atmosphere. The reactions constants are computed using the following expression:

$\;\log K_{p,i}=A\log\left(T/1000\right)+B/T+C+DT+ET^{2}$ with the constants *A*, *B*, *C*, *D*, and *E* given in Table 1.

We find ourselves with a set of 11 nonlinear equations with 11 unknowns which are solved using Newton Raphson iteration. A detailed description of the resolution algorithm can be found in [13]. The method was implemented using a modified Matlab script provided by [16] to insert new subroutines of the fuel injection and pulverization parameters as well as the biodiesel constant and the double Wiebe function of heat release rate.

## 4. Results and Discussion

For validation purposes the two models were implemented and compared with experimental results from [17], the engine characteristics are given in Table 2 where fuel injection timing refers to the actual crankshaft angle at which fuel starts coming out of the nozzle. Two sets of calculation were performed, one for diesel fuel and the second for biodiesel fuel. The characteristics of diesel and biodiesel fuel were taken from [17, 18] for method 1. To simulate biodiesel combustion for method 2 we used methyl butanoate thermodynamic data as surrogate [14].

The experimental imep (indicative mean effective pressure) was not given in the experimental work reported by [17], it was therefore evaluated using the experimental pressure trace by integrating the area under the *P*-*V* diagram using the trapezoidal rule, and the formula used was then

(25) $$\begin{array}{c} \text{imep}=\overset{V_{f}}{\underset{V_{s}}{\int }}\frac{Pdv}{V_{d}}=\overset{n}{\underset{i=1}{\sum }}\frac{\left(v_{i+1}-v_{i}\right)\left(P_{i}+P_{i+1}\right)}{2} \end{array}$$

with *V*_*d*_ being the displaced volume of the engine, in m^3^.

**Figure 1:**
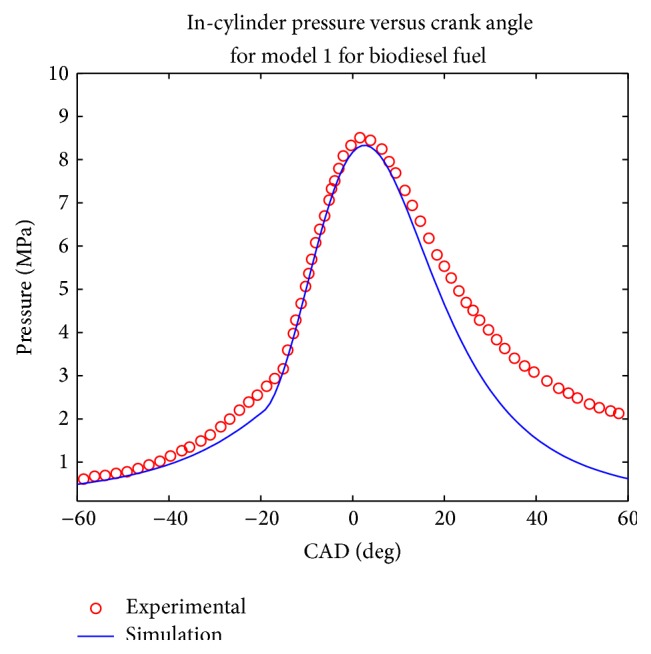
Comparison of computed and experimental pressure trace for model 1 for biodiesel fuel.

**Figure 2:**
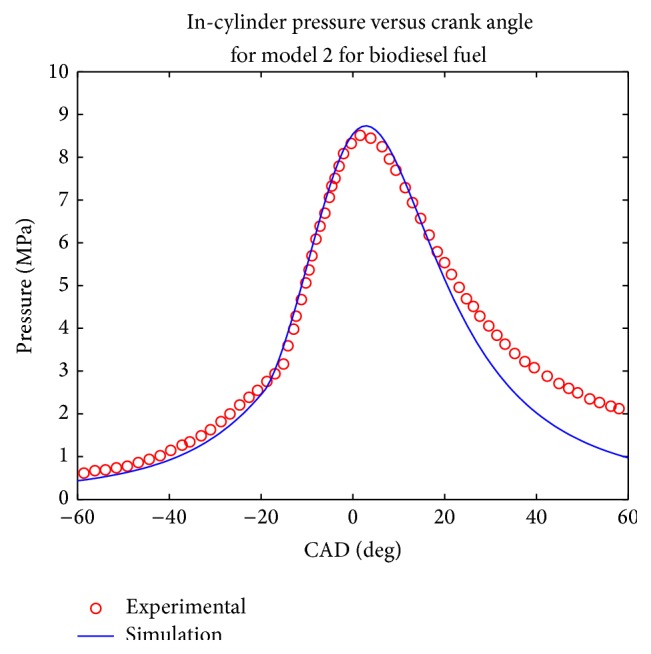
Comparison of computed and experimental pressure trace for model 2 for biodiesel fuel.

**Figure 3:**
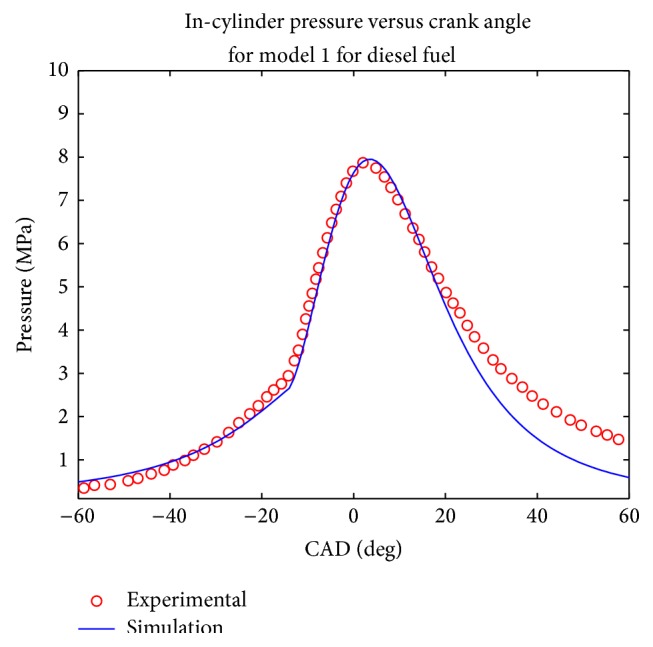
Comparison of computed and experimental pressure trace for model 1 for diesel fuel.

**Figure 4:**
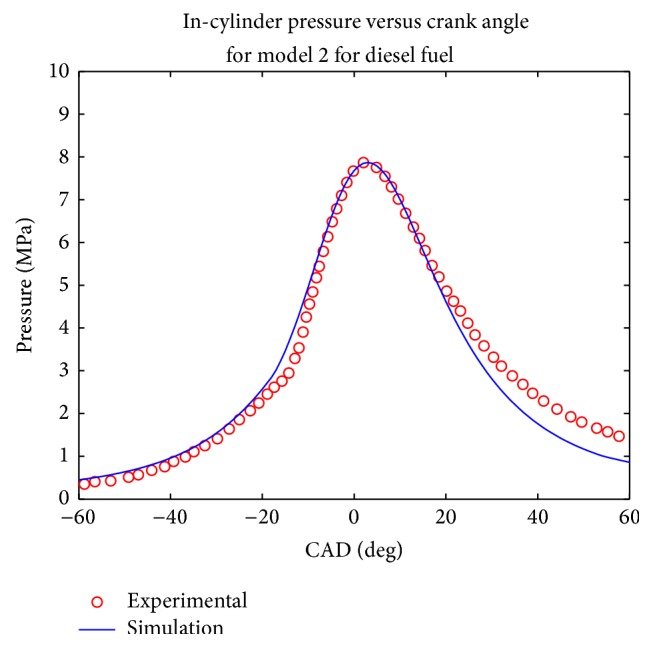
Comparison of computed and experimental pressure trace for model 2 for diesel fuel.

Simulations were computed at rated engine speed and the pressure plot was made and compared with the experimental results (Figures 1, 2, 3, and 4). Both models evaluated the combustion duration at 72 CAD and 87 CAD, respectively, for diesel and biodiesel fuel. This can be explained by the fact the viscosity and density of biodiesel are significantly higher than that of conventional diesel, which tends to generate pulverized droplets with higher mean diameter that tend to burn slower.

The pressure traces obtained from the two model show that they reproduce well the in cylinder pressure for both type of fuels in the given engine configuration. It can be seen that the maximum pressure decreases when biodiesel fuel is used for the two simulations, which is equally the trend observed experimentally and in other works such as [19–22]. This was expected since biodiesel fuel has higher oxygen content; the combustion coefficient tends to be higher in that case. Another trend observed in the pressure trace is that the cylinder pressure tends to be under predicted during the exhaust phase, especially for biodiesel fuel; this could be explained by the experimental conditions uncertainty.

The values of computed parameters are given in Table 3 in terms of maximum pressure, imep, maximum pressure occurrence, and ignition delay. In order to have an evaluation of the accuracy of both model, the mean relative error was calculated according to the four parameters given above, it was then observed that the first model gave a mean relative error of 18% while the second one had given a mean relative error of 11%.

## 5. Computation Speed

Another assessment made for the two models was the speed of computation; the first model was computed in 6 seconds, while the second took about 40 to 45 second to run completely on 2 Ghz dual core Pentium personal computer.

With this assessment, we can say that the first model could be suitable for real time engine control while the second could be used for engine's design and also emission evaluation since it computes in cylinder pressure by determining combustion species states at each time step. Another development that could be made for the second model can be the implementation of the Zeldovich mechanism [1, 11] to compute nitric oxide emissions.

## 6. Conclusion

The aim of the present study was to develop and implement two different models for diesel engine fuelled on biodiesel based on tabulated chemistry. The two models showed good predictability of engine performance, with the second approach giving a better accuracy. The first approach was faster to compute the cylinder pressure than the second and was found more suitable for engine monitoring and control while the second one was found better suited for engine design and emission prediction.

## Figures and Tables

**Table 1:** Olikara and Borman constants.

	*A*	*B*	*C*	*D*	*E*
*K*_*p*,1_	0.432168	−0.112464 × 10^5^	0.267269 × 10^1^	−0.745744 × 10^−4^	0.242484 × 10^−8^
*K*_*p*,2_	0.310805	−0.129540 × 10^5^	0.321779 × 10^1^	−0.738336 × 10^−4^	0.344645 × 10^−8^
*K*_*p*,3_	−0.141784	−0.213308 × 10^4^	0.853461	0.355015 × 10^−4^	−0.310227 × 10^−8^
*K*_*p*,4_	0.150879 × 10^−1^	−0.470959 × 10^4^	0.646096	0.272805 × 10^−5^	−0.154444 × 10^−8^
*K*_*p*,5_	−0.752364	0.124210 × 10^5^	−0.260286 × 10^1^	0.259556 × 10^−3^	−0.162687 × 10^−7^
*K*_*p*,6_	−0.415302 × 10^−2^	0.148627 × 10^5^	−0.475746 × 10^1^	0.124699 × 10^−3^	−0.900227 × 10^−8^

**Table 2:** Engine specifications.

Number	Particular	Specifications
1	Make	Kirloskar oil engine
2	Model	DAF 8
3	Rated brake power (kW)	6
4	Rated speed (rpm)	1500
5	Number of cylinder	1
6	Bore × stroke (mm)	95 × 110
7	Compression ratio	17.5 : 1
8	Fuel injection timing	23°

**Table 3:** Comparison of simulated engine's combustion performances with experimental data.

	Diesel			Biodiesel		
	Model 1	Model 2	Experiment	Model 1	Model 2	Experiment
Max pressure (MPa)	7.948	7.87	7.87	8.37	8.73	8.53
Imep (MPa)	0.52	0.47	0.6	0.522	0.6	0.7
Maximum pressure occurrence (CA after TDC)	4	3	5.8	3	3	5
Ignition delay (CAD)	8.69	8.69	8.5	4.37	4.37	4.3

**Table 4:** Krieger and Borman coefficients.

		0	1	2	3	4	5	6
*a*			0.692	3.917*e* − 5	5.29*e* − 8	−2.29*e* − 11	2.7758*e* − 17	
*b*		3049.33	−0.057	−9.5*e* − 5	2.153*e* − 8	−2*e* − 12		
*c*_*u*_	2.32584							
*c*_*r*_	0.004186							
*d*		10.41066	7.85125		−3.71257			
*e*		−15001	−15838		9613			
*f*		−0.10329	−0.38656		0.154226	−14.763	118.27	14.503
*r*		−0.2977	11.98	−25442	−0.4354			

## Appendix

For more details see Table 4.
